# Digital Microfluidics-Powered Real-Time Monitoring of Isothermal DNA Amplification of Cancer Biomarker

**DOI:** 10.3390/bios12040201

**Published:** 2022-03-28

**Authors:** Beatriz Jorge Coelho, Bruno Veigas, Luís Bettencourt, Hugo Águas, Elvira Fortunato, Rodrigo Martins, Pedro V. Baptista, Rui Igreja

**Affiliations:** 1 Department of Materials Science, School of Science and Technology, NOVA University of Lisbon and CEMOP/UNINOVA, Campus de Caparica, 2829-516 Caparica, Portugal; bj.coelho@campus.fct.unl.pt (B.J.C.); l.bettencourt@campus.fct.unl.pt (L.B.); hma@fct.unl.pt (H.Á.); emf@fct.unl.pt (E.F.); rfpm@fct.unl.pt (R.M.); 2 UCIBIO, I4HB, Life Sciences Department, School of Science and Technology, NOVA University of Lisbon, Campus de Caparica, 2829-516 Caparica, Portugal; 3 AlmaScience, Campus da Caparica, 2829-519 Caparica, Portugal; bruno.veigas@almascience.pt

**Keywords:** digital microfluidics, loop-mediated isothermal amplification, real-time nucleic acid amplification monitoring, fluorescence detection, cancer biomarker

## Abstract

We introduce a digital microfluidics (DMF) platform specifically designed to perform a loop-mediated isothermal amplification (LAMP) of DNA and applied it to a real-time amplification to monitor a cancer biomarker, *c-Myc* (associated to 40% of all human tumors), using fluorescence microscopy. We demonstrate the full manipulation of the sample and reagents on the DMF platform, resulting in the successful amplification of 90 pg of the target DNA (0.5 ng/µL) in less than one hour. Furthermore, we test the efficiency of an innovative mixing strategy in DMF by employing two mixing methodologies onto the DMF droplets—low frequency AC (alternating current) actuation as well as back-and-forth droplet motion—which allows for improved fluorescence readouts. Fluorophore bleaching effects are minimized through on-chip sample partitioning by DMF processes and sequential droplet irradiation. Finally, LAMP reactions require only 2 µL volume droplets, which represents a 10-fold volume reduction in comparison to benchtop LAMP.

## 1. Introduction

Cancer is currently one of the leading causes of death worldwide according to the World Health Organization [1], with over 19 million new cases and almost 10 million deaths in 2020, as estimated by the International Agency for Research on Cancer [2]. Point-of-care (POC) devices are relevant agents in early cancer detection [3], performing specific tests near the patient, with a much smaller delay between testing and receiving the final result [4,5] in comparison to centralized facilities [6,7]. Such devices support the identification of cancer biomarkers [8,9] as the *c-Myc* oncogene, known as a “master regulator” [10] due to its involvement in cell growth, proliferation, and metabolism, which cumulatively intervene in tumorigenesis initiation and expansion [10,11,12,13,14]. Moreover, the overexpression of *c-Myc* has been associated with a large percentage (40%) of tumors [10,11], which further increases the clinical relevance of this oncogene. Digital microfluidics (DMF) arises as a promising base technology for POC devices, namely in the field of cancer biomarker detection, allowing for the precise control of individual low-volume droplets (nano- to micro-liters) over an electrode array [15,16,17,18,19,20,21]. Having such a high degree of freedom, droplets can perform any routine assigned by the user, according to electrode disposition [16,22,23,24,25,26,27]. In particular, in the most common DMF configuration (closed configuration), droplets are sandwiched between two plates, thus enabling fluidic operations: dispensing, mixing, merging, or splitting. In this configuration, droplet motion is typically achieved through electrowetting-on-dielectric phenomenon, whereby an electric field is applied between operating electrodes and the ground electrode (usually the top plate), forcing charge redistribution within the droplet, and enabling droplets to move from a non-actuated (OFF) to an actuated (ON) electrode [28]. 

In the present work, we describe the design, construction, and application of a DMF platform for automating biological assays, particularly isothermal nucleic acid amplification detection for screening cancer biomarker *c-Myc*. We specifically highlight two novelties: DMF-based real-time isothermal amplification monitoring of a cancer biomarker and a dual strategy for on-chip reagent mixing. Loop-mediated isothermal amplification (LAMP) is the chosen amplification methodology, considering its outstanding robustness, specificity, and sensibility [29,30,31,32]. Moreover, as an isothermal strategy, LAMP eliminates intricate thermal cycling procedures and subsequently allows lower energy consumption. Herein, we combine DMF with standard fluorescence microscopy to achieve the real-time monitoring of isothermal DNA amplification. We have successfully designed and fabricated a thin, light, and moveable DMF platform, which can be easily integrated with a fluorescence microscope. Special attention is directed toward the heating system included within the DMF platform, based on a transparent thin film heating element, which ensures stable heating. Due to the low volume of droplets carried by DMF, the laminar flow is predominant, meaning that merging two or more droplets does not immediately result in mixing of droplet content or uniformization of molecule distribution along the merged droplet [33,34,35,36]. Several strategies have been developed to address this issue, namely AC signal frequency tuning [35,36,37,38,39] or specific electrode path architectures [33,34,40]. Herein, we include active mixing in our DMF-LAMP routine through a combination of two mixing strategies: low AC frequency mixing and straightforward back-and-forth motion, to ensure uniform and efficient mixing of the LAMP master mix with test samples. We further compare the efficiency of mixing LAMP reagents inside our DMF device versus mixing reagents off-chip by vortex agitation before performing the LAMP reaction on-chip.

Other DMF devices have been developed for DNA amplification purposes [22], however the large majority relies on thermal cycling for PCR (Polymerase Chain Reaction) and are dedicated to the detection of pathogens and not cancer biomarkers. Moreover, for both isothermal and non-isothermal methodologies, on-chip reagent mixing is not investigated [41,42,43,44,45]. Moreover, this DMF platform provides key advantages when compared to our previous platform [23], namely the following: (i) compatibility with optical/fluorescence microscopy, which more importantly allows for real-time LAMP reaction monitoring; (ii) performing multiple reactions simultaneously; (iii) significantly lower area occupation of the DMF platform (approximately 7 times lower volume); and (iv) enhanced temperature control. We also designed a specialized software for droplet control and monitoring, which is adaptable to any electrode path architecture. Such advancements to the previous system [23] allow for the easy automatization of the LAMP reaction protocol, as well as real-time reaction monitoring, thus placing this platform on track for the sample-to-answer POC category. The produced setup enables the LAMP-amplification of a small amount of the target DNA (90 pg, 0.5 ng/µL) and further distinctions between amplifying and non-amplifying samples in under 1 h. Moreover, the proposed on-chip dual mixing strategy enables an increase in fluorescence readouts during LAMP in comparison to off-chip reagent mixing.

## 2. Materials and Methods

### 2.1. DMF Chip Design and Production

Patterns for electrode paths and reservoirs were designed using Adobe llustrator^®^ software (Adobe Inc., San Jose, CA, USA) and transferred onto film photomasks (JD Photodata, Hitchin, UK) for photolithography. Glass substrates were cleaned with acetone and isopropyl alcohol baths under ultrasound (model Sonorex Super—Bandelin electronic GmbH, Berlin, Germany), at 15 min each, and rinsed with ultrapure water. Substrates were then heated at 180 °C to ensure evaporation of any water molecules on the surface, and after brief cooldown, they were coated with AZ ECI 3012 1.2 µm grade photoresist (MicroChemicals, Ulm, Germany) by spin-coating (Suss Labspin6, Suss MicroTec SE, Garching, Germany) at 2000 rpm for 10 s, followed by 4000 rpm for 20 s. Pre-baking was then performed at 115 °C for 75 s. Exposure was carried out under UV radiation on a Suss MA6 UV mask aligner (Suss MicroTec SE, Garching, Germany) for 8 s. Following exposure, substrates were post-baked at 115 °C for 35 s and further developed in AZ726 MIF developer (MicroChemicals, Ulm, Germany) also for 35 s. Afterwards, a 200 nm chromium layer was deposited via a home-made electron beam deposition system (substrate heating at 100 °C), and the final conductor patterns were achieved after lift off with acetone. A 2 µm layer of Parylene C (Specialty Coating Systems, Indianapolis, IN, USA; CAS 28804-46-8) was then deposited over the chromium pattern (Specialty Coating Systems Labcoater^®^-PDS 2010, Indianapolis, IN, USA). Lastly, a solution of 0.6% wt/wt of Teflon^®^ AF 1600 in Fluorinert FC-40 (DuPont, Wilmington, DE, USA) was spin-coated over the dielectric at 1000 rpm for 30 s (Model WS-650MZ-23NPP–Laurell, North Wales, PA, USA) to achieve a 50 nm thickness and post-baked at 160 °C for 10 min. For the top plates, ITO-coated glass was used, and it was also covered by a Teflon^®^ hydrophobic layer through the same process described above. The 180 µm gap between top and bottom plates was set by polyimide tape (Pro-Power Farnell, Leeds, UK), and both plates were sealed together by simply using nail polish dried at 55 °C for 10 min. Since the nail polish was placed around the reservoirs, this layer also isolates the central area of the chip.

### 2.2. DMF Platform: Electrode Pattern and DMF Device Support System

**Figure 1:**
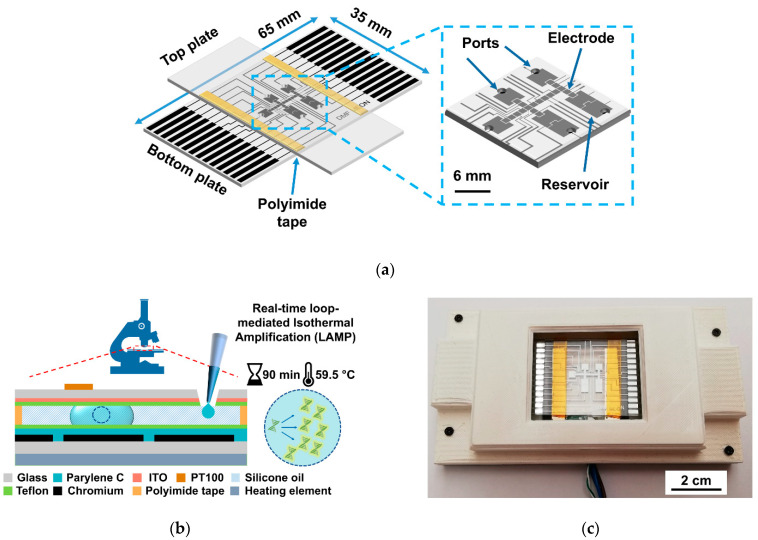
Multiview of the DMF device and assemblage. (**a**) Schematic view of the DMF device, with an inset zoom of the electrode and reservoir area; (**b**) cross section of a DMF device (not to scale), evidencing all the composing layers and real-time LAMP reaction parameters; (**c**) DMF device embedded onto the 3D-printed support system (top plate omitted for better visualization).

The developed DMF chip is based on the closed configuration whereby the bottom plate includes voltage-actuating electrodes, and the top plate allocates the common ground. Figure 1a,b illustrate the developed DMF device, both overall and in cross-section. In this device, the bottom plate electrode pattern comprised seven large reservoirs that were inter-connected by an array of nineteen smaller electrodes (Figure 1a). Such a design allows all fluidic operations required for two simultaneous LAMP reactions (see Section 2.6), and the further integration of a transparent thin-film heating element directly below the bottom plate enables the device heating necessary for DNA amplification (Figure 1b). Holes drilled on the top plate allow reagent insertions on the DMF device and reaction product retrieval for subsequent output confirmation by gel electrophoresis (Figure 1a,b). To accommodate both the DMF chip and heating element, as well as to establish a connection between the DMF electrodes and the droplet control system, a custom DMF support system was produced (Figure 1c). This support system was designed with a 3D modelling software (Fusion 360^®^, Autodesk, San Rafael, CA, USA) and all parts were printed with an Ultimaker 2+^®^ 3D printer (Ultimaker B.V., Utrecht, Netherlands) in polylactic acid (PLA), with the exception of the thin film resistor support, which was printed in acrylonitrile butadiene styrene (ABS), since this polymer can withstand higher temperatures. The height of the support system is merely 1.9 cm for easy integration with a standard optical microscope. Figure S1 (Supplementary Materials) contains detailed information on the design and purpose of all parts constituting this support system, as well as an illustration of all hardware required for chip operation and optical microscope integration for fluorescence measurements. Supplementary Video S1 further provides a demonstration of all fluidic operations enabled by the DMF chip.

### 2.3. DMF Droplet Driving System

The driving system is fully controlled by a specific software developed in-house (see Supplementary Information S2), which is connected to an Arduino Mega 2560 microcontroller board. The Arduino board translates input information from the software to digital output signals and is further transmitted to the HVSU (high voltage switching unit). The HVSU receives an amplified AC signal (40 Vrms, 5 kHz or 0.5 kHz) and according to the commands provided by the Arduino board, it activates/deactivates the corresponding electrodes on the chip by enabling/disabling the connection to the AC signal, respectively. A block diagram containing all entities and connections of the droplet driving system may be found in Supplementary Information S2.

### 2.4. Temperature Control System

**Figure 2:**
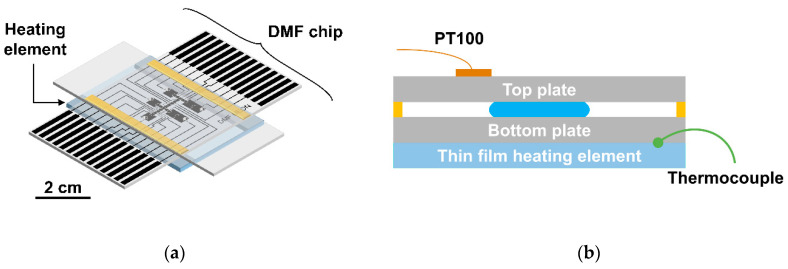
(**a**) Schematic view of the DMF chip (as depicted in Figure 1) and the thin film heating element placed directly below the bottom plate for on-chip LAMP reactions; (**b**) Cross section of the same configuration, evidencing the location of the temperature sensors, namely the PT100 sensor on the top plate and the thermocouple on the heating element. Image not to scale.

The temperature control system includes an ITO (Indium-Tin-Oxide)-coated glass, which generates heat, and a PT100 temperature sensor (RS PRO PT100 class B 2 platinum chip—2 mm diameter, RS Components, Corby, UK), which controls the feedback system for temperature regulation. Essentially, 30 × 35 mm^2^ pieces were cut from commercially available ITO-coated glass to fit within the thin film resistor support. A 66 nm Titanium-Gold (6 nm of Titanium for glass adhesion and 60 nm of Gold for improving electric conduction properties) layer was then deposited on opposite ends of the ITO-coated glass pieces by electron beam deposition as contacts. Following this, multifilament cable wires were bonded to Ti-Au contacts by means of conductive silver ink (PELCO^®^ Conductive Silver Paint, Ted Pella. Inc., Redding, CA, USA) and high-temperature epoxy glue (Ceys high temperature contact glue, Ceys, Barcelona, Spain), which were left to dry for 24 h. For heating, the assembled thin film resistor was placed downwards on the respective support so that the non-coated part would be in contact with the DMF device. The bottom plate of the DMF chip was placed over the thin film resistor and a PT100 sensor was bound to the top plate of the chip near the reaction region. An additional temperature sensor (RS PRO Type K Thermocouple, RS Components, Corby, UK) was glued to the thin film resistor, thus allowing for a better monitoring of the temperature gradient across the entire DMF chip. Figure 2 illustrates the integration of the temperature sensors with the DMF chip for on-chip LAMP reactions.

Temperature control is based on a feedback system, where power applied to the heating element is controlled by a MOSFET transistor. The PT100 sensor information is fed to an Arduino-based implementation of a PID (proportional integral derivative) code that correspondingly adjusts the output power in the MOSFET. Supplementary Information S3 includes a detailed description of the temperature distribution along the thin film heating element with feedback from the PT100 temperature sensor.

### 2.5. Loop-Mediated Isothermal (LAMP) Reaction

LAMP reactions, either on- or off-chip, were performed as previously described [23] using a master mix consisting of 1× of ThermoPol^®^ reaction buffer (New England Biolabs, Ipswich, MA, USA), 1 M betaine (Sigma–Aldrich, St. Louis, MO, USA), 4 mM magnesium sulfate (MgSO_4_, New England Biolabs, Ipswich, MA, USA), 0.8 µM of both FIP (Forward Inner Primer) and BIP (Backward Inner Primer), 0.4 mM of dNTP mix (ThermoFisher Scientific, Waltham, MA, USA), 0.2 µM of both Forward Primer (FP) and Backward Primer (BP), 1.5× EvaGreen^®^ (Biotium, Fremont, CA, USA), and 8 U of Bst DNA polymerase (large fragment; New England Biolabs, Ipswich, MA, USA). The concentration of EvaGreen^®^ was optimized to fit the operation range (see Supplementary Information S4), and after analyzing the optimization results, a final concentration of 1.5× EvaGreen^®^ was selected. For the positive controls (amplifying samples), a DNA fragment containing the target sequence derived from the *c-Myc* gene was added to the master mix for a final concentration of 0.5 ng/µL, whereas for the negative control (non-amplifying samples), PCR-grade water was added instead. All primers were obtained from Stab Vida (Caparica, Portugal), and the sequences are described in Supplementary Information S5.

### 2.6. On-Chip LAMP Protocol

For on-chip LAMP reactions, three strips of polyimide tape were placed on each side of the bottom plate (180 µm spacing), and nail polish was then applied around the electrode area. After positioning the top plate over the bottom plate, the DMF chip was heated at 55 °C for 10 min to dry the nail polish and to seal the electrode area. Before sample addition, each chip was filled with 5 cSt silicone oil (Sigma-Aldrich, St. Louis, MO, USA) that was previously degassed in a vacuum for at least 3 h. Sealing the electrode area within the DMF chip, as well as degassing the silicone oil filler for a minimum of 3 h, produced excellent results against droplet evaporation.

**Figure 3:**
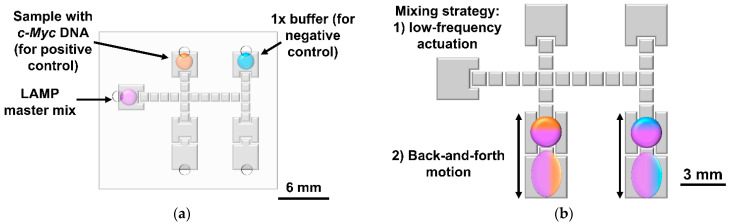

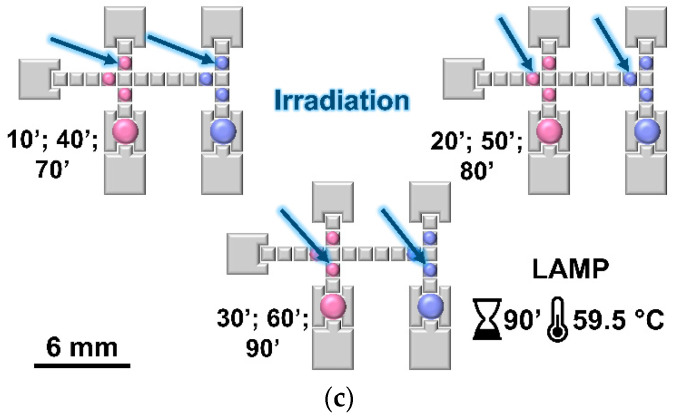
Steps required for on-chip LAMP amplification with on-chip reagent mixing. Droplets containing the LAMP master mix, *c-Myc* DNA, and 1× reaction buffer were inserted onto the respective reservoirs (**a**). The LAMP master mix is then moved towards the two reservoirs at the bottom of the chip (1.62 µL per large droplet, achieved with nine small 180 nL droplets. For the positive control, a small droplet of DNA solution was merged with one partition of the LAMP master mix, whereas for the negative control, a small droplet of 1× reaction buffer was merged with the second partition of the LAMP master mix. Mixing is performed by low AC frequency actuation followed by back-and-forth droplet motion (**b**), and three droplets are withdrawn from each control and moved towards irradiation sites (**c**). The DMF chip was then heated for a 59.5 °C setpoint measured at the top plate for 90 min, and droplets were sequentially irradiated (**c**). Note that for Figure 3b,c, the top plate was omitted for better visualization.

For on-chip reagent mixing, three different solutions were prepared: (1) LAMP master mix; (2) DNA solution consisting of the target *c-Myc* gene fragment resuspended in 1× ThermoPol^®^ reaction buffer; and (3) standard 1× ThermoPol^®^ Reaction buffer. All three solutions were added to separate reservoirs, as illustrated by Figure 3a. The LAMP master mix was divided into two partitions (each containing nine droplets of approximately 180 nL each, considering the electrode area and top/bottom plate spacing), which were moved towards the reaction areas, and one droplet from both the 1× reaction buffer and the solution containing the DNA target was added to the master mix partitions. Excess liquid from all three solutions remaining on the reservoirs was removed to prevent air bubble formation due to device heating for LAMP, and degassed silicone oil was used to cover the inlets as to avert the entrance of air. The mix of the DNA solution and the buffer solution droplets with the LAMP master mix partitions was performed by switching the frequency of the actuation signal to 0.5 Hz for 6 s, followed by back-and-forth motions of the droplets between the larger reservoirs at the bottom part of the chip (Figure 3b). The resulting fully mixed droplets were further subdivided into three smaller droplets (Figure 3c) as to prevent the eventual photobleaching of the fluorophore due to continuous irradiations of the reaction mixture. The DMF platform (chip and support system) was then placed under a fluorescence microscope (model BX51—Olympus, Tokyo, Japan) and heated for a 59.5 °C setpoint measured at the top plate for 90 min. During the LAMP reaction, every 10 min, one small droplet from each control was irradiated (Figure 3c), and the emission captured by the microscope detector is stored in image format. With this protocol, during the 90 min of reaction, each droplet is irradiated for a maximum of three times, which greatly contributes to reducing photobleaching resulting from the use of non-specialized equipment, such as a real-time cycler. For droplet motions, an AC signal with 40 V_RMS_ and 5 kHz was used, whereas during a LAMP reaction, the signal amplitude was reduced to 20 V_RMS_ to prevent device degradation. Finally, the endpoint LAMP products (i.e., all droplets from each reaction) were pulled together by DMF, removed from the device, and resolved in 1% agarose gels in 1× TAE buffer (Tris-Acetate-EDTA) at 70 V for 90 min.

For off-chip reagent mixing, positive and negative controls were prepared as previously described (see Section 2.5) and reagents were mixed by vortex mixing. Both controls were then added to the DMF chip, and the protocol followed the procedures listed above, except for the steps required for on-chip mixing. Supplementary Information S6 provides a detailed description of the DMF procedure for off-chip reagent mixing.

## 3. Results and Discussion

### 3.1. Temperature Monitoring for On-Chip LAMP

**Figure 4:**
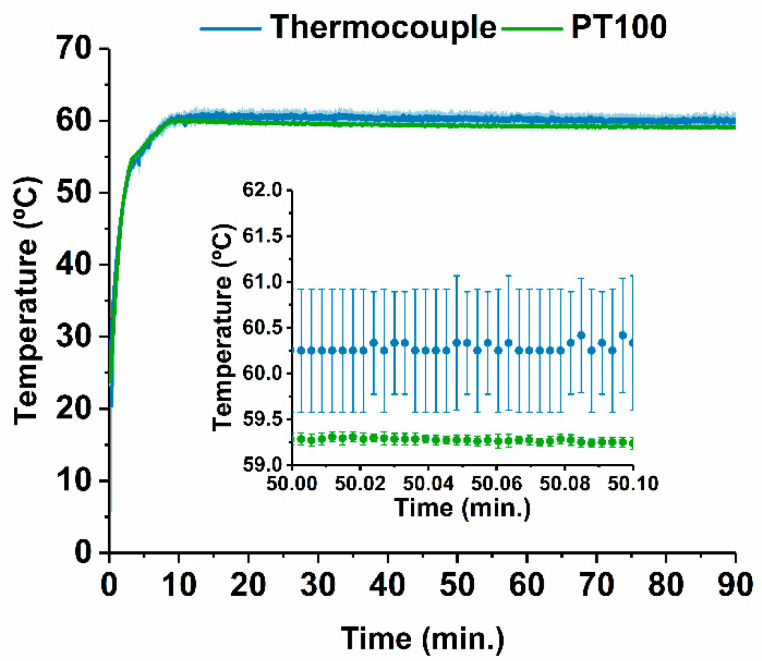
Average temperature measured on two separate locations of the DMF system by two distinct temperature sensors, a PT100 and a thermocouple, for three trials. On the inset, the region around the setpoint temperature is enlarged for better visualization. Error bars correspond to the 90% confidence interval for a total of three experiments.

Considering that LAMP occurs between 60 °C and 65 °C, the setpoint temperature for the PT100 sensor was adjusted to 59.5 °C, thus ensuring that the temperature gradient within the reaction droplet falls between the above-mentioned range. As an extra control measure, a thermocouple was attached to the edge of the thin film resistor. Resorting to this setup, the PID parameters were then adjusted to prevent temperature overshoot, thus preventing the degradation of the LAMP reagents, and to achieve a low steady-state error, thus maintaining the reaction temperature constant. Figure 4 represents the average temperature measured by both temperature sensors in a total of three experiments.

As observed in Figure 4, the temperature quickly increased to 55 °C, after which the temperature steadily reaches a setpoint of 59.5 °C in approximately 4 min with a good steady-state error on the top plate level (maximum deviation of 0.9 °C from 10 min to 90 min).

### 3.2. On-Chip LAMP

**Figure 5:**
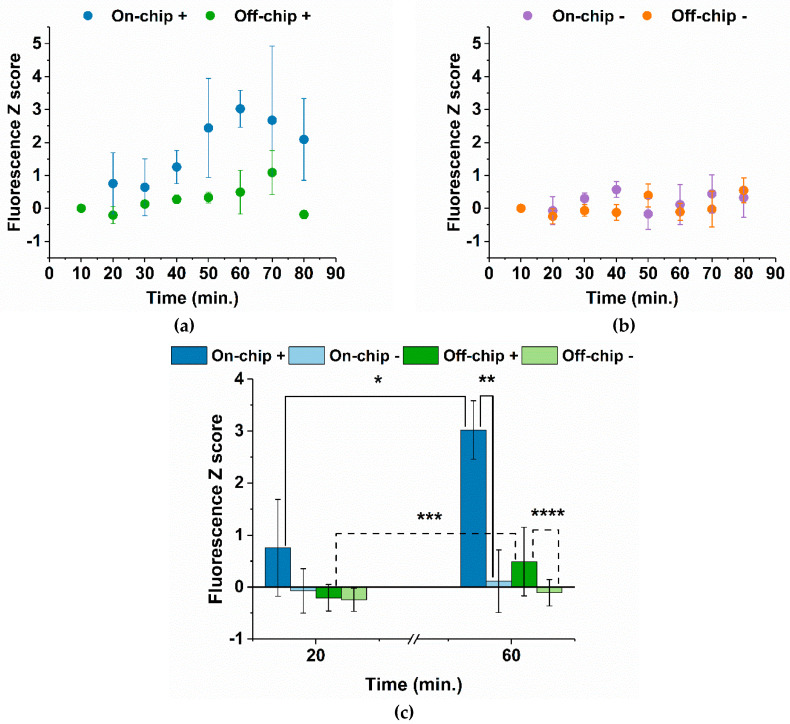
Fluorescence Z score analysis for LAMP reactions. (**a**) Z scores for amplifying “+” samples, where reagents were mixed either on-chip or off-chip. (**b**) Z scores for non-amplifying “-” samples, also comparing reagent mixing on-chip and off-chip. (**c**) Fluorescence Z scores for early and final stages of DMF-LAMP reactions, concerning all tested conditions, namely amplifying and non-amplifying samples, as well as off-chip and on-chip reagent mixing. Statistical analysis was performed by using one-way ANOVA (analysis of variance) and means comparison by the Tukey method. * *p* ≤ 0.05, ** *p* ≤ 0.003, *** *p* ≤ 0.3, and **** *p* ≤ 0.2 for a significance level of 0.05. In all cases, error bars correspond to the 90% confidence interval of a minimum 4 experiments for each time frame.

After determining the ideal conditions for fluorescence measurements, DMF-LAMP reactions were performed with reagent mixing on-chip and off-chip. In both cases, for each 10 min, one positive droplet and one negative droplet were quickly exposed, and the resulting emission was captured as an image. At the end of the LAMP reaction, all images were processed by an in-house designed software, which essentially divides the image into two color channels (green for the fluorescent droplet and black for the background) following an Otsu segmentation algorithm and subsequently determines the median fluorescence intensity for both the droplet and background, subtracting the latter from the first. Each experiment was performed at least four times. The Z score for fluorescence was then determined according to Equation (1):

(1) $$Z\;\mathrm{score}=\frac{X-X_{i}}{SD_{X_{i}}}$$

where *X* is the current fluorescence measurement, *X_i_* is the first fluorescence measurement, and *SD_Xi_* is the standard deviation for the first fluorescence measurement. The Z score was chosen as the primary statistical treatment for the fluorescence data, as it illustrates the capacity of our system to detect changes in fluorescence intensity by normalizing the data to the standard deviation of the baseline (i.e., the first measurement at 10 min). Figure 5 summarizes the results achieved for fluorescence measurements.

Figure 5a shows that on-chip mixing yields a greater variation in fluorescence for amplifying samples and, therefore, possesses a superior capacity to detect the production of double-stranded DNA as a result of LAMP amplification. With on-chip mixing, DNA amplification is clearly visible from 40 min onwards, with the fluorescence amplification plateau reached at 60 min. As for off-chip mixing, the largest increase in fluorescence occurs from 60 min to 70 min, at which point the plateau is reached. Cumulatively, lower Z scores were observed for off-chip mixing, influenced by greater fluctuations in the initial fluorescence measurements, possibly suggesting an increase in mixing efficiency for on-chip versus its off-chip counterpart. Data in Figure 5b demonstrate that fluorescence in non-amplifying samples remains mostly unchanged as expected, with Z scores close to 0, regardless of on-chip or off-chip mixing. Still, some background noise of the system is observed, probably due to the formation of primer dimers resulting from the initial molecular steps of the LAMP reaction, as the EvaGreen^®^ fluorophore intercalates into double-stranded DNA [46] (see Supplementary Information S7). Supplementary Information S8 provides additional information regarding relative fluorescence. Figure 5c outlines the fluorescence Z scores of all tested conditions (on-chip and off-chip mixing, amplifying “+”, and non-amplifying “-”) for the early and final stages of DMF-LAMP reactions. The DMF platform allows for a distinction between amplifying and non-amplifying samples, whether mixing is performed on-chip or off-chip. In both cases, fluorescence increasingly deviates from the baseline; nevertheless, the said deviation is larger for on-chip reagent mixing, as previously observed. Data presented on Figure 5 suggest that the proposed DMF platform is suitable for performing real-time LAMP using only 0.5 ng/µL (or 90 pg) of the *c-Myc* proto-oncogene template. Moreover, the proposed dual on-chip mixing strategy based on low frequency signal actuation and back-and-forth motion for mixing the LAMP master mix with test sample seems to promote a relevant decrease in initial fluorescence variability, as reflected by overall larger Z scores. This suggests that the proposed system seems to be able to detect smaller changes in fluorescence when the LAMP master mix and the sample are mixed on-chip. A further examination of the mixing mechanisms specifically associated with the low-frequency actuation and the back-and-forth motion is currently being pursued within the group in order to understand the impact that each strategy has on the final mixing result. Nevertheless, the dual mixing strategy has, thus, proven to be successful in overcoming laminar flow, a significant constraint for mixing in microfluidic systems [36,39]. The simplified setup still requires some optimization as to increase the dynamic range of the signal acquisition, mainly by reducing the background noise for the non-amplifying reactions. Once this signal-to-noise ratio is improved, the proposed system might be of use for nucleic acid detection and semi-quantification (comparison) between samples. Furthermore, we envision the integration of said nucleic acid detection onto a portable DMF platform equipped with fluorescence detection capability. This could be achieved through the miniaturization of all DMF-operating hardware (i.e., signal wave generator, signal amplifier, power source, voltage control hardware, and temperature control hardware—see Supplementary Information S1, Figure S1.3), following the lead of other portable DMF systems such as PortaDrop [47], LampPort [48], or the DropBot DB3-120 kit [49].

## 4. Conclusions

In this work, we designed and produced a DMF platform for isothermal amplification of cancer biomarker *c-Myc* and real-time reaction monitoring by using fluorescence. The developed DMF chip was designed to perform simultaneous amplifications of positive and negative LAMP controls, while fitting an optical microscope, thus enabling fluorescence measurements for the reactions. Fluorophore photobleaching resulting from the use of non-specialized equipment was surpassed by dividing each reaction droplet into three smaller droplets irradiated in turn so that each droplet would only be irradiated three times. Furthermore, the heating system allows stable temperature maintenance, with a maximum error of 0.9 °C at the steady-state region (from 10 min to 90 min). Moreover, we have successfully developed a novel mixing strategy for DMF, involving both low-frequency signal actuation (0.5 Hz, 40 V_rms_) and back-an-forth motion. This strategy was validated by using real-time LAMP amplification on the *c-Myc* oncogene, and it resulted in a larger increase in fluorescence readouts for amplifying samples during LAMP, as well as an overall decrease in fluorescence fluctuation. Such developments enable isothermal DNA amplification detection of 0.5 ng/µL or 90 pg of the *c-Myc* cancer biomarker within one hour, with a clear distinction between amplified and non-amplified samples. This work paves the way for future developments envisioning the simultaneous amplification of two genes for gene expression analysis. Once these issues have been addressed, we shall be able to follow suit with the validation of the proposed platform using clinically relevant samples, i.e., comparing the amplification profiles of *c-Myc* and a housekeeping gene for semi-quantification of the biomarker. Moreover, the future miniaturization of the DMF operation hardware and integration of on-chip fluorescence detection will enable a truly field-deployable system for real-time cancer biomarker monitoring.

## Data Availability

The data presented in this study are available upon request from the corresponding author.

## Supplementary Materials

The following Supplementary Information can be downloaded at: https://www.mdpi.com/article/10.3390/bios12040201/s1. S1: DMF support system components and detailed functionality description. S2: DMF droplet control software and hardware. S3: Temperature distribution along the heating element. S4: Optimization of EvaGreen^®^ concentration. S5: Primer sequences used for all LAMP reactions. S6: DMF protocol for off-chip reagent mixing. S7: Agarose gel electrophoresis of positive vs negative controls. S8: Relative fluorescence for on- and off-chip reagent mixing. Supplementary Video S1: Demonstration of fluidic operations on the DMF chip. Supplementary Video S2: Example of a DMF-based LAMP procedure.
