# Supplementary material for: Digital Microfluidics-Powered Real-Time Monitoring of Isothermal DNA Amplification of Cancer Biomarker
Source: Biosensors (Basel). 2022 Mar 28;12(4):201. doi: 10.3390/bios12040201 (PMC9028060; doi:10.3390/bios12040201)
Supplement: Supplementary file 1 [file biosensors-12-00201-s001.zip › biosensors-1640800-supplementary.pdf]

biosensors

Article

Digital Microfluidics-powered Real-time Monitoring of Isothermal DNA Amplification of Cancer Biomarker

Beatriz Jorge Coelho [1,2], Bruno Veigas [3], Luís Bettencourt [1], Hugo Águas [1], Elvira Fortunato [1], Rodrigo Martins [1], Pedro V. Baptista [2,*] and Rui Igreja [1,*]

1 Department of Materials Science, School of Science and Technology, NOVA University of Lisbon and CEMOP/UNINOVA, Campus de Caparica, Caparica 2829-516, Portugal; bj.coelho@campus.fct.unl.pt (B.J.C.); l.bettencourt@campus.fct.unl.pt (L.B.); hma@fct.unl.pt (H.Á.); emf@fct.unl.pt (E.F.); rfpm@fct.unl.pt (R.M.)
2 UCIBIO, I4HB, Life Sciences Department, School of Science and Technology, NOVA University of Lisbon, Campus de Caparica, Caparica 2829-516, Portugal
3 AlmaScience, Campus da Caparica, Caparica 2829-519, Portugal; bruno.veigas@almascience.pt (B.V.)
* Correspondence: pmvb@fct.unl.pt (P.V.B.); rni@fct.unl.pt (R.I.)

Supplementary Information

S1: DMF support system components and detailed functionality description

The DMF support system consists of two major parts, the cover and the base, and both 3D-printed. The base is further subdivided into three components: 1) ground connector base, 2) ground connector, 3) printed circuit board (PCB) and outer connection support, and 4) thin film resistor support. Figure S1.1 represents a deconstructed 3D model for cover and base of the DMF support system, evidencing all components.

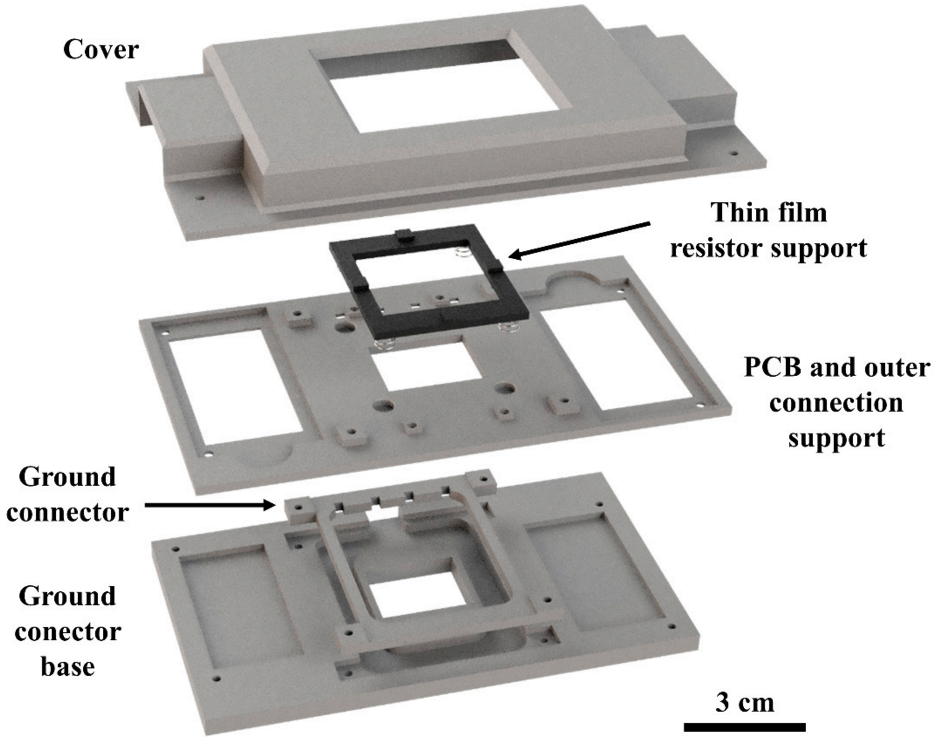

Figure S1. 1: 3D model of the support system for DMF chips, evidencing all separate components, by fitting order.

The ground connector base simply provides a casing for wire protection and fixation, while maintaining a flat, straight foundation for the entire platform. The ground connector is covered in conductive silver ink (PELCO® Conductive Silver Paint, Ted Pella. Inc., Redding, CA, USA) and essentially ensures the short-circuit of all the conductive pins providing electrical contact with the ITO layer from the top plate (see Figure S1.2) and also to connect the aforementioned elements to the ground. Considering that the LAMP assay requires heating (in this case, via a thin film resistor), a physical support was specifically designed to hold the heater and it was kept as close to the DMF chip as possible, while providing an air gap to dissipate heat beneath the heater, thus protecting the integrity of surrounding materials. Finally, the PCB and outer connection support is yet another separate piece (see Figure S1.2), which includes room to incorporate a specially designed PCB that allows connection between the DMF chip (through 2 PCB edgecard connectors – 28 contacts, 2.54 mm pitch; Weald Electronics, Horsham, UK) and the driving hardware (through 2 PCB connectors – 16 contacts, 2.54 mm pitch, male; Harting, Espelkamp, Germany). This component also includes dedicated areas to fit four springs suspending the thin film resistor support, as well as four pogo pins (P70-2100045R—5.5 mm free height, Harwin, Portsmouth, UK) for connecting the top plate to the ground, exerting an upward force onto the top plate of the DMF chip. The cover protects both the DMF chip and electronic circuitry, includes an open window so that fluorescence may be captured, and also lodges another four pogo pins, which are slightly shorter than the previous ones (P70-21000045R—3.6 mm free height, Harwin, Portsmouth, UK), that exert downward force onto the top plate. The eight pogo pins (gour on the cover and gour on the base) fixate the top plate of the DMF chip, and since they all contain internal springs, the top plate can move just enough to respect the gap between plates, defined by polyimide tape (180 µm). All parts are inter-connected through a set of eight screws. Figure S1.2 illustrates the fully assembled base, including the DMF chip and related exterior connections.

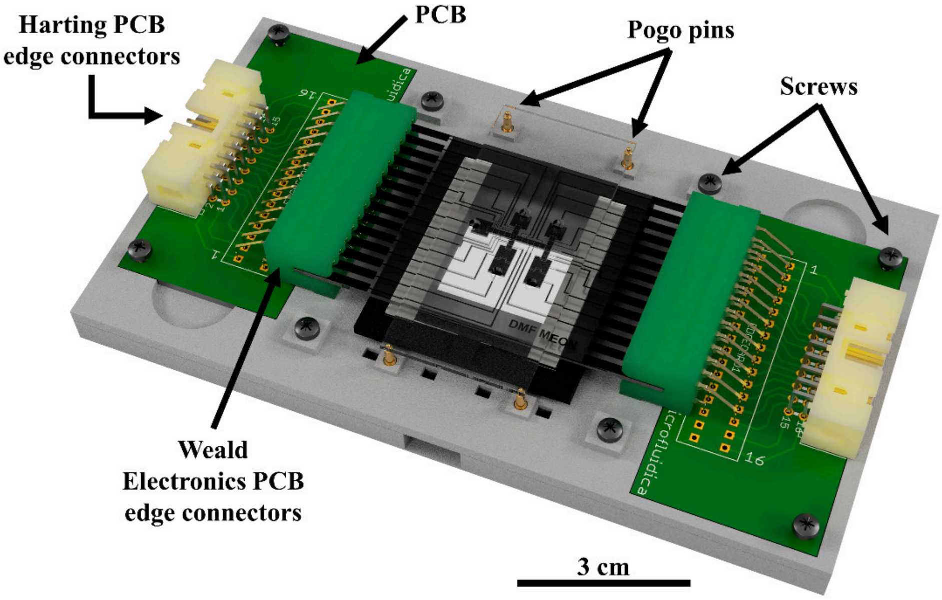

(**a**)

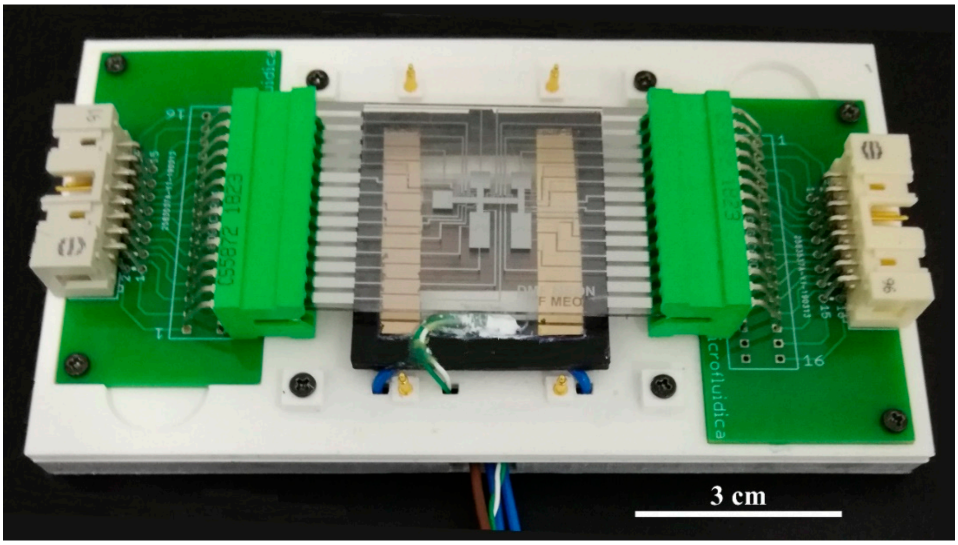

(**b**)

Figure S1. 2: 3D illustration (a) and photograph (b) of the uncovered DMF support system, evidencing connections from the pads of the chip to PCB edge connectors and outer-support system connectors, mediated by a PCB.

Base and cover measure 13.2 cm x 7.3 cm, with a total thickness of only 1.9 cm, thus fitting under a fluorescence microscope. Figure S1.3 displays all the hardware required to operate the DMF devices, evidencing the integration of the DMF platform onto the optical microscope.

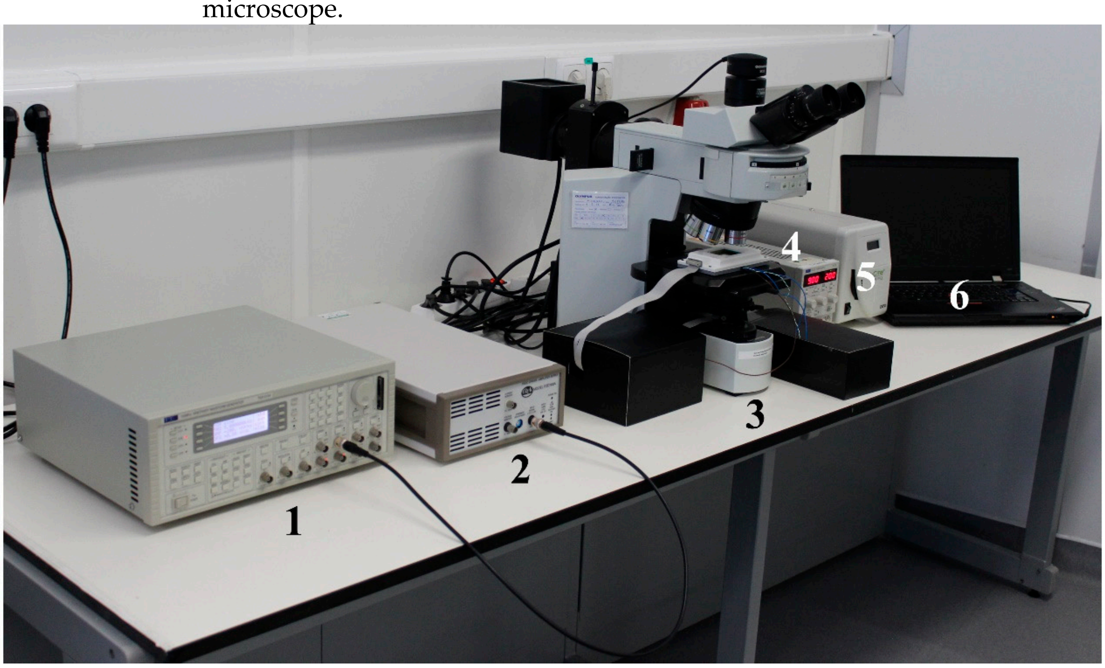

(**a**)

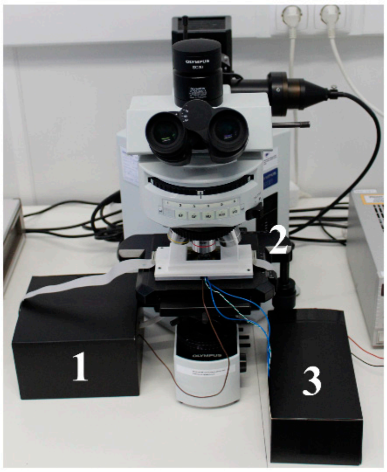

(**b**)

Figure S1. 3: a) General view of the operation hardware for the DMF platform: 1—signal wave generator; 2—signal amplifier; 3—optical microscope setup; 4—power source; 5—fluorescence light source; 6—control laptop. b) Zoom on the optical microscope setup required for fluorescence measurements: 1—voltage control box; 2—DMF platform (DMF chip and support system); 3—temperature control box.

S2: DMF droplet control software and hardware

Electrode switching for droplet control is fully automatic, relying on a software specifically designed for this purpose. This software was implemented through the Python programming language, which includes a specialized module for Graphical User Interface (GUI) implementation: Tkinter. This module allows for the straightforward creation of operation menus and facilitates communication with any external hardware (cameras, Arduino boards, other electronic equipment). Figure S2.1 illustrates the user interface of the software.

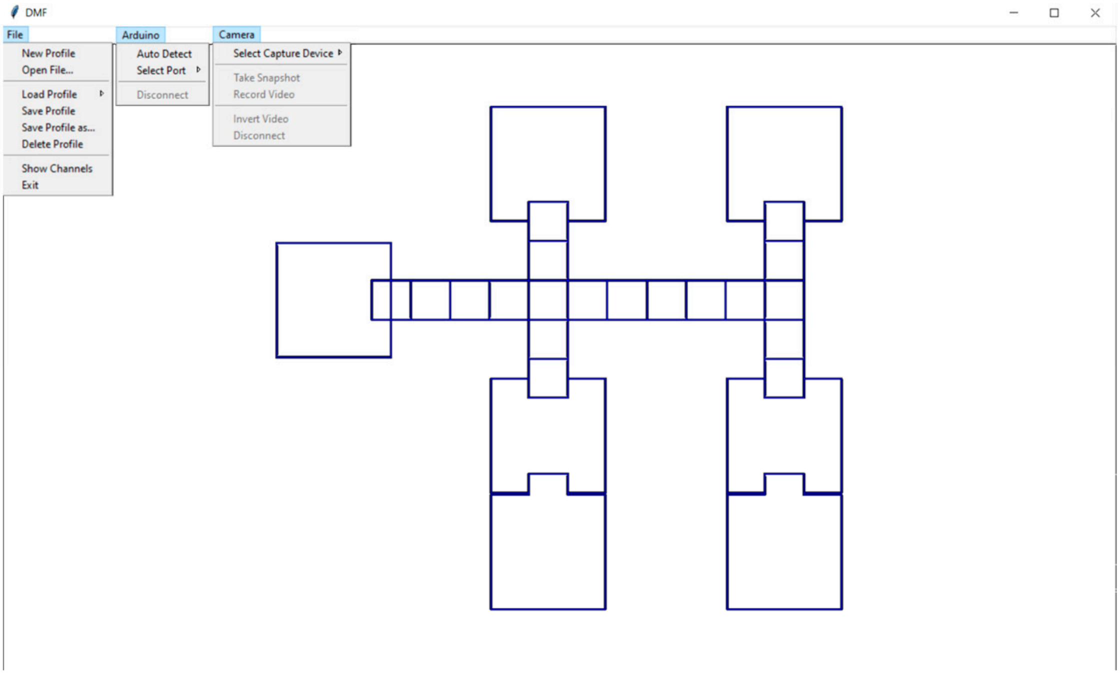

Figure S2. 1: Edited screenshot of the software with the configured device profile to display all available options in the top menu bar.

As observed in Figure S2.1, the software enables three different main operations: 1) profile uploading, which allows the user to create an .svg file containing any bottom plate electrode configuration. The software assumes designed shapes as addressable entities, which may be turned ON or OFF as desired; 2) board detection, enabling communication with an Arduino board, which in turn will control the high voltage switching unit and ultimately allow for switching ON/OFF any set of electrodes; 3) camera control, which enables selection of any USB-connectable camera device, as well as video and snapshot capturing.

The developed software is the primary means of communication with the DMF chip. Following an ON command at the computer software, the Arduino control board will transmit a high-state signal (5V) to the high voltage switching unit, which will apply an external signal (generated by a wave generator and further amplified) to the selected DMF chip electrode. Figure S2.2 presents the block diagram for the above-mentioned process.

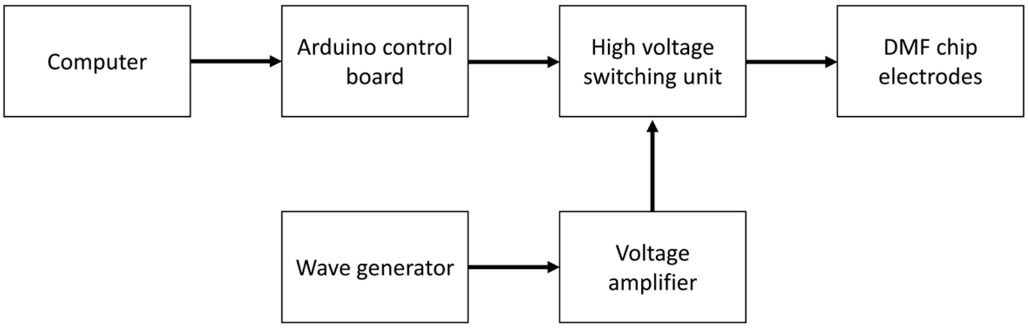

Figure S2. 2: Block diagram representation of the multi-component DMF droplet driving system, evidencing connections between distinct components.

S3: Temperature distribution along the heating element

It is important to understand the temperature distribution along the thin film heating element, thus ensuring that temperature is equally distributed along the areas where LAMP reactions will occur. For this purpose, the PT100 sensor was bound directly to the thin film heating element for temperature control, and an infrared camera (PYROVIEW 380L compact+, DIAS Infrared Systems, Dresden, Germany) was used to film the thermal profile of the resistor during heating, from room temperature up to 65 °C. A higher temperature than necessary was tested, considering that heat will be dissipated from the measuring point to the real reaction area. Figure S3.1 shows a compilation of frames evidencing the evolution of temperature within the thin film heating element.

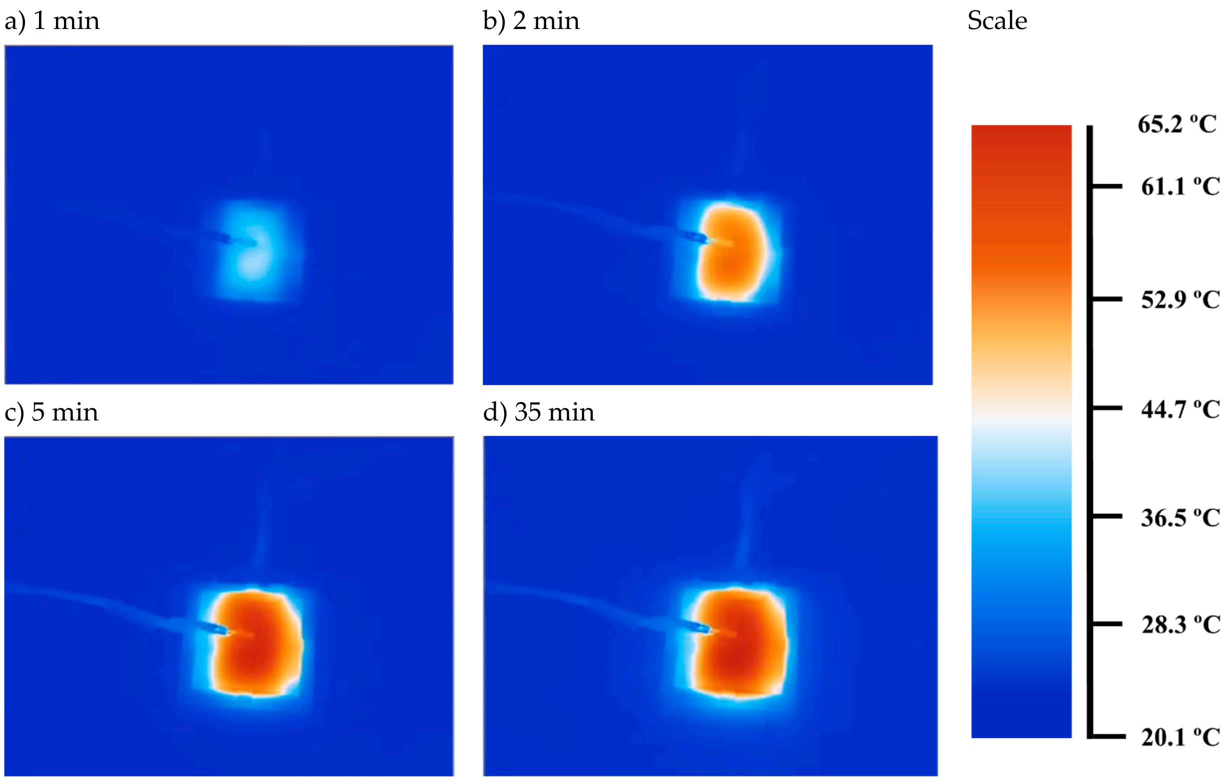

Figure S3. 1: Video frames acquired through the PYROSOFT software, companion to the PYROVIEW camera family, displaying the temperature distribution along the thin film heating element, from room temperature to a 65 °C setpoint.

The temperature initially rises in the middle section of thin film resistor (Figure S3.1 a) and b) and rapidly spreads along the entire area, reaching the setpoint (around 65 °C) in about 5 min. The area directly beneath the reaction region of the DMF chip is uniformly heated; therefore, the thin film resistor is a viable option for on-chip nucleic acid amplification reactions. Temperature measured by the thermal camera was also confirmed by a PT100 sensor placed on top of the heating element, directly beneath the area where LAMP reactions will occur (see Figure S3.2).

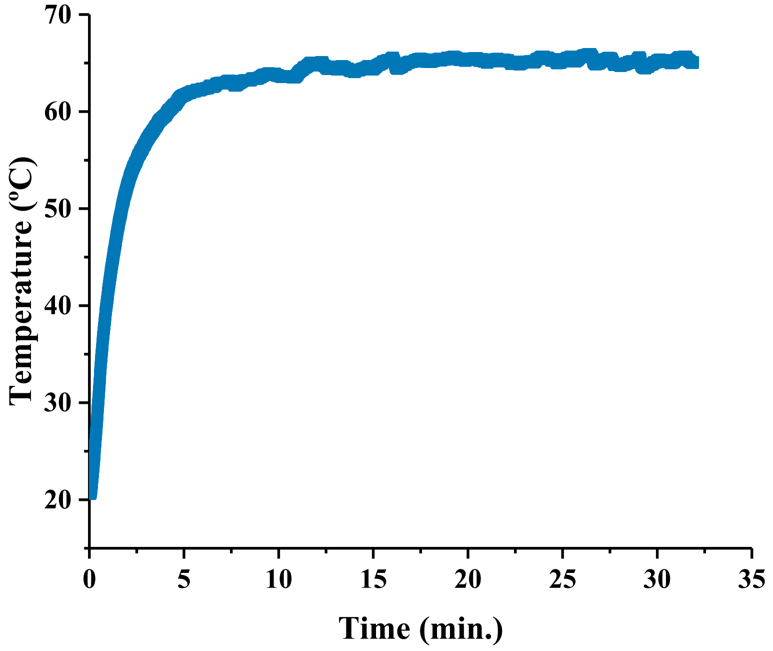

Figure S3. 2: Temperature measured by the PT100 sensor placed directly on top of the thin film resistor.

S4: Optimization of EvaGreen® concentration

Prior to on-chip reactions, LAMP conditions were optimized for best fluorescence readout. Thus, 1 µL droplets of both positive and negative LAMP controls were sandwiched between dummy bottom and top plates (180 µm height), mimicking on-chip conditions. This setup was further placed under a fluorescence microscope (model BX51—Olympus, Tokyo, Japan), and the samples were irradiated by an EXFO X-Cite Series 120 Q lamp (Excelitas, Waltham, MA, USA). Both excitation and emission were processed through a U-MWB2 (Olympus, Tokyo, Japan) blue broadband filter cube. Three different concentrations of EvaGreen® fluorophore were tested (1x, 1.5x and 2x) by adding a suitable volume at the end of the reaction time. This procedure was repeated in triplicate for all conditions. Figure S4.1 represents the output results of the abovementioned experiment.

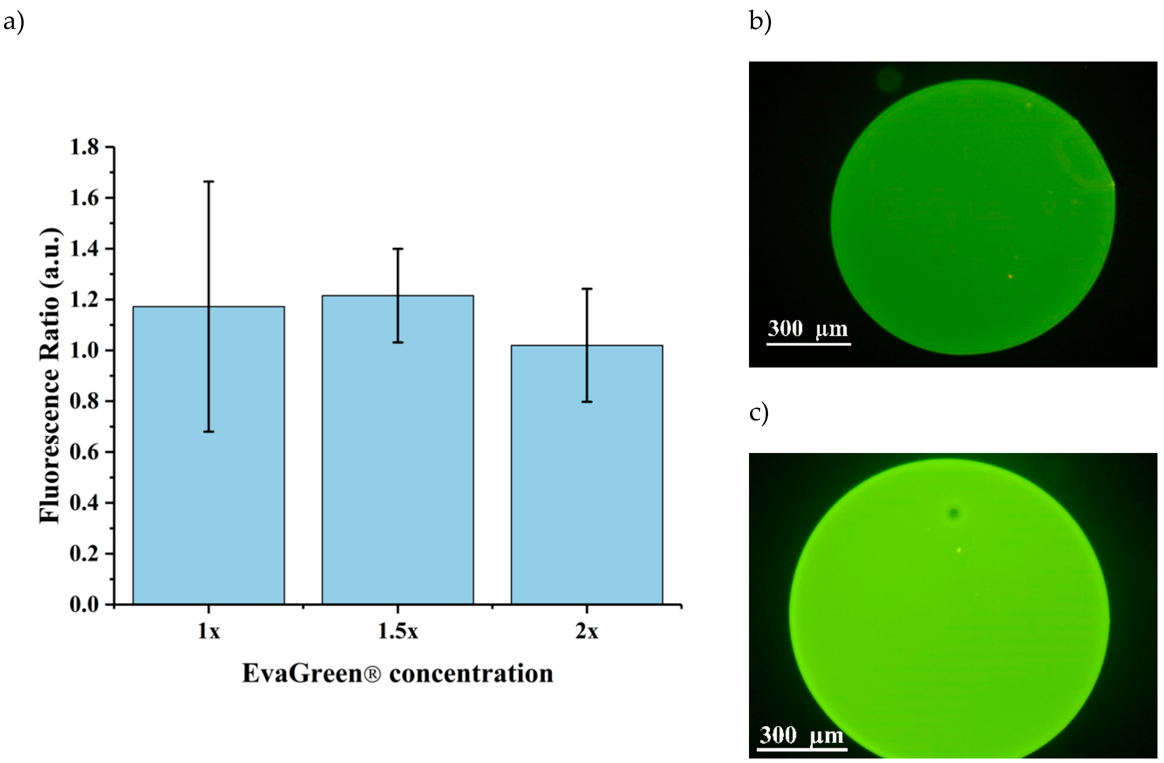

Figure S4. 1: a) Fluorescence ratio between amplifying and non-amplifying samples[1] for multiple EvaGreen® initial concentrations (1x, 1.5x or 2x). Error bars correspond to the confidence interval at 90% for each case for a total of three experiments. Example of non-amplifying (b) and amplifying (c) samples.

Amplifying samples generally achieve higher fluorescence intensity than non-amplifying samples regardless of EvaGreen® concentration. However, EvaGreen® at 1.5x cumulatively presents a reasonable difference in fluorescence between amplification and non-amplification, as well as a less significant error; thus, this was the chosen condition.

S5: Primer sequences used for all LAMP reactions

Table S5. 1: Primer sequences used for the LAMP-amplification of the *c-Myc* oncogene.

PRIMER	SEQUENCE (5′- 3′)
FP	TCTGAAGAGGACTTGTTGC
BP	TTCAGTCTCAAGACTCAGC
FIP	CTTTTCCTTACGCACAAGAGTTCC-GGAAACGACGAGAACAG
BIP	ACGATTCCTTCTAACAGAAATGTCC-CAAGGTTGTGAGGTTGCA

S6: DMF protocol for off-chip reagent mixing

In this case, positive and negative controls were prepared as described in section *Loop-mediated isothermal (LAMP) reaction* of the main text, and the reagents were mixed by using vortex mixing. Subsequently, positive and negative LAMP control samples were inserted onto the chip via access orifices on the top plate (Figure S6.1 a) and moved toward

[1] The fluorescence ration between amplifying and non-amplifying samples is determined by $\frac{Amplifying\ average\ fluorescence}{Non-amplifying\ average\ fluorescence}$

the reaction region via digital processes (Figure S6.1 b). Three smaller droplets were then split from the positive and negative control droplets (Figure S6.1 c). After correct positioning of both smaller and larger droplets, the DMF platform (chip and support system) was placed under a fluorescence microscope (model BX51—Olympus, Tokyo, Japan) and heated for a 59.5 °C setpoint measured at the top plate for 90 min. During the LAMP reaction, every 10 min, one small droplet from each control is irradiated (Figure S6.1 d) and the emission was captured by the microscope detector. Irradiation conditions are similar to those referred in the main text, as well as droplet actuation parameters. Finally, the endpoint LAMP products (i.e., all droplets from each reaction) were pulled together by DMF, removed from the device (Figure S6.1 e and f). and submitted to agarose gel electrophoresis analysis. Supplementary Video 2 provides an example of such a protocol.

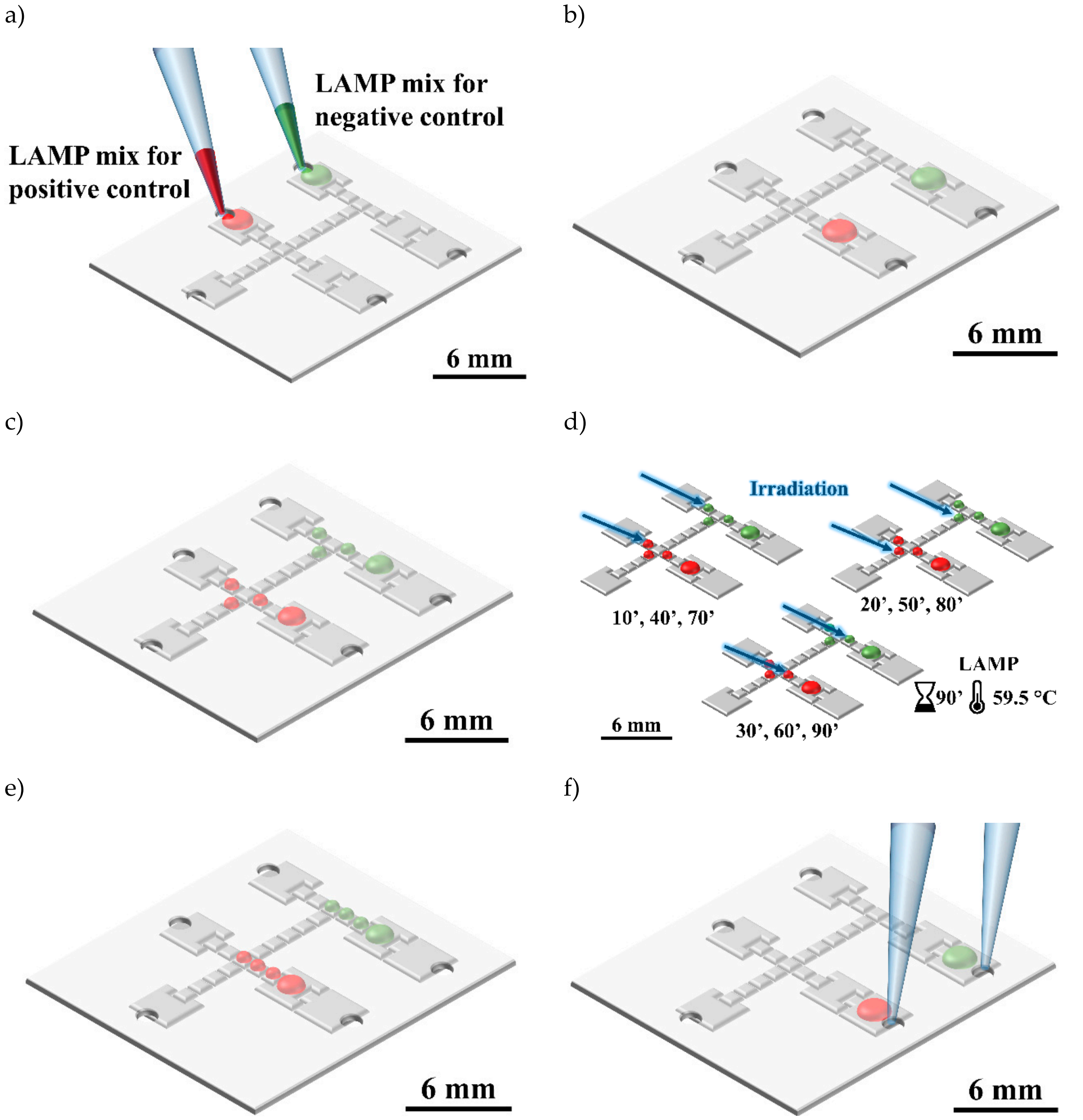

Figure S6. 1: Steps required for on-device LAMP amplification of sample DNA, with off-chip reagent mixing. Firstly, droplets containing the positive and negative controls are moved towards the reaction areas (a-b). Secondly, three small droplets are withdrawn from each control and placed along the electrode paths (c). The DMF chip is then heated with 59.5 °C measured at the top plate, for 90 min and the smaller droplets are sequentially irradiated (d). Finally, the smaller droplets are merged with the original ones, as to be removed from the device (e-f).

S7: Agarose gel electrophoresis of positive vs negative controls

For on-chip LAMP reactions, background noise in the form of negative control fluorescence was detected by the proposed DMF system. Considering that the working mechanism of the EvaGreen® fluorophore consists of binding to double-stranded DNA, fluorescence for the negative control could be due to the formation of primer dimers, which may be identified by agarose gel electrophoresis as low-sized strands (Figure S7.1).

a)

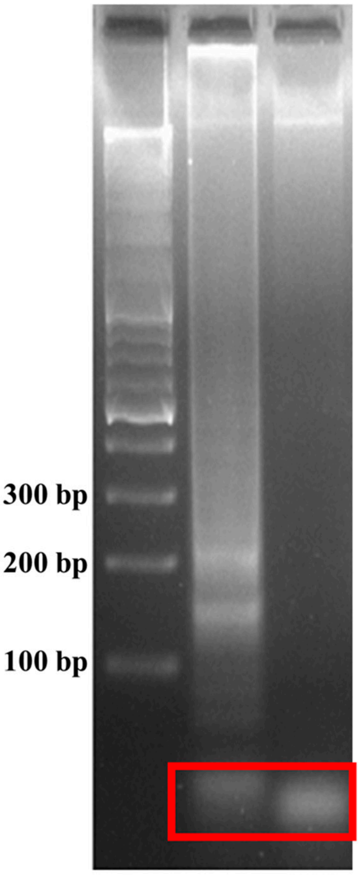

b)

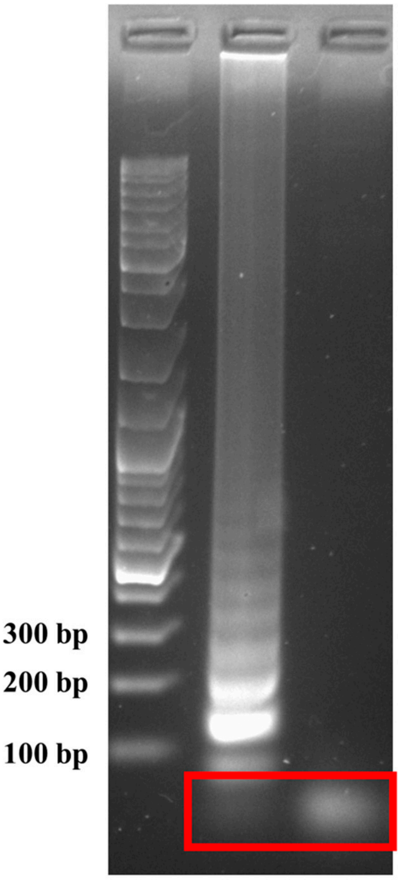

Figure S7. 1: Examples of gel electrophoresis conducted for two distinct on-chip LAMP experiments, where primer dimers are visible (highlighted in red). For both trials (a and b), the lane order, from left to right, is as follows: DNA ladder ǀ positive control ǀ negative control.

S8: Relative fluorescence for on- and off-chip reagent mixing

Figure S8.1 illustrates the relative fluorescence achieved with both on-chip and off-chip mixing of the LAMP master mix with the DNA sample.

a) b)

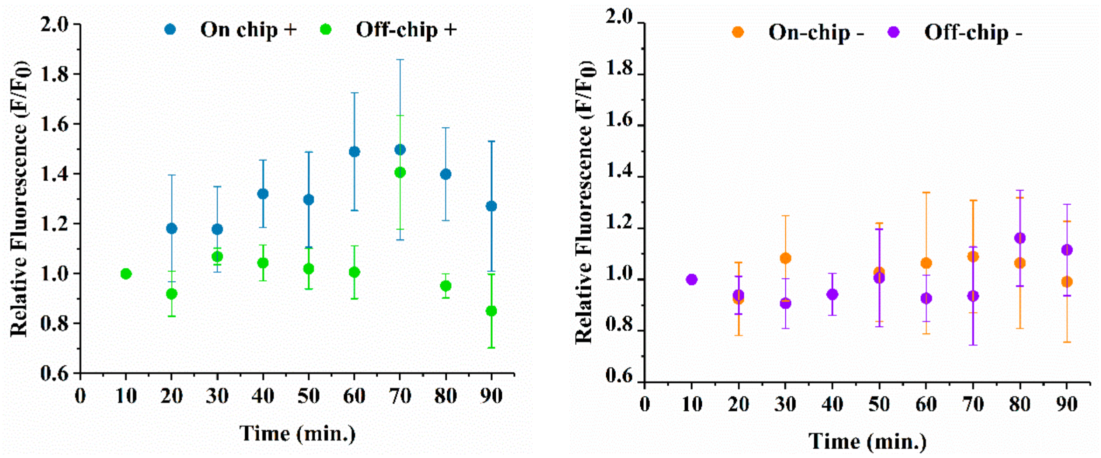

Figure S8. 1: Relative fluorescence (normalized to the initial measurement at 10 min) in LAMP reactions, for both amplifying "+" (a) and non-amplifying "-" (b) samples, where reagents were mixed either on-chip or off-chip. Error bars correspond to the 90% confidence interval of a minimum 4 experiments for each time frame.

Regarding amplifying samples, similarly to the information withdrawn from Z scores at the main text, the analysis of the relative fluorescence reveals that target DNA amplification is detected from 40 min onwards with on-chip mixing, whereas for off-chip mixing, amplification is only detectable from 60 min to 70 min. Moreover, for non-amplifying samples (mixed either on- or off-chip), fluorescence does not significantly increase during the reaction time, as would be expected. However, a slight increment in fluorescence is visible at 80 min and 90 min, which is attributed to background noise generated by the formation of primer dimers (see section S7) to which the fluorophore can bind.
